# Induction of Endogenous Antimicrobial Peptides to Prevent or Treat Oral Infection and Inflammation

**DOI:** 10.3390/antibiotics12020361

**Published:** 2023-02-09

**Authors:** Kimberly A. Morio, Robert H. Sternowski, Kim A. Brogden

**Affiliations:** 1 Apex Endodontics, Hiawatha, IA 52233, USA; 2 Softronics, Co., Ltd., Marion, IA 52302, USA; 3 College of Dentistry, The University of Iowa, Iowa City, IA 52242, USA

**Keywords:** antimicrobial peptide, AMP, defensin, cathelicidin, micronutrients, nutrients, macronutrients, ultraviolet, UVB, UVC, inflammation, pain, healing

## Abstract

Antibiotics are often used to treat oral infections. Unfortunately, excessive antibiotic use can adversely alter oral microbiomes and promote the development of antibiotic-resistant microorganisms, which can be difficult to treat. An alternate approach could be to induce the local transcription and expression of endogenous oral antimicrobial peptides (AMPs). To assess the feasibility and benefits of this approach, we conducted literature searches to identify (i) the AMPs expressed in the oral cavity; (ii) the methods used to induce endogenous AMP expression; and (iii) the roles that expressed AMPs may have in regulating oral inflammation, immunity, healing, and pain. Search results identified human neutrophil peptides (HNP), human beta defensins (HBD), and cathelicidin AMP (*CAMP*) gene product LL-37 as prominent AMPs expressed by oral cells and tissues. HNP, HBD, and LL-37 expression can be induced by micronutrients (trace elements, elements, and vitamins), nutrients, macronutrients (mono-, di-, and polysaccharides, amino acids, pyropeptides, proteins, and fatty acids), proinflammatory agonists, thyroid hormones, and exposure to ultraviolet (UV) irradiation, red light, or near infrared radiation (NIR). Localized AMP expression can help reduce infection, inflammation, and pain and help oral tissues heal. The use of a specific inducer depends upon the overall objective. Inducing the expression of AMPs through beneficial foods would be suitable for long-term health protection. Additionally, the specialized metabolites or concentrated extracts that are utilized as dosage forms would maintain the oral and intestinal microbiome composition and control oral and intestinal infections. Inducing AMP expression using irradiation methodologies would be applicable to a specific oral treatment area in addition to controlling local infections while regulating inflammatory and healing processes.

## 1. Introduction

Antibiotics are used throughout all disciplines of dentistry for the treatment of both soft and hard tissue infections [1]. These treatments include infections associated with the pulp and periapical tissues, oral mucosal and oropharyngeal tissues, and oral lesions in immunocompromised patients, especially during radiotherapy and chemotherapy. Antibiotics are also used for prophylaxis against infections in individuals with joint replacements and heart defects prior to teeth cleaning or surgical procedures [1]. While the use of antibiotics has clear benefits, their overuse can alter the composition of the oral microbiomes, create niches on mucosal surfaces for opportunistic microorganisms such as *Candida* species to colonize, promote the development of antibiotic-resistant microorganisms, and result in complications associated with hypersensitivity reactions and allergic disorders [1,2,3].

Antimicrobial peptides (AMPs) have been recently proposed as alternatives to antibiotics for treating oral infections [4]. However, there have been concerns related to producing and using AMPs as small-peptide therapeutics. AMP molecule stability, toxicity, elimination half-life, and clearance are obstacles that have hindered the development of AMPs [5]. There can also be difficulties in correctly folding the complex three-dimensional structures seen in larger AMPs [6,7]. Finally, the antimicrobial activities of AMPs are modest in host environments. AMPs do not withstand the physiological conditions in the host environment, including the activity of host proteinases, differences in site pH, differences in ionic conditions, and the presence of polyanionic glycosaminoglycans [8,9].

Host-directed therapy (HDT) is a concept proposed to boost innate immune mechanisms for controlling antibiotic-resistant microorganisms [10,11]. Included in HDT is the use of inducers or “elicitors” to express endogenous AMPs for treating infection and inflammation [6,10,12,13,14,15,16,17,18].

In this study, we hypothesized that this approach could be used to induce AMP transcription and expression in the oral cavity. We started by conducting an initial literature search to identify AMPs expressed in the oral cavity. Human neutrophil peptide (HNP)-1, -2, and -3 defensins [19], the human beta defensins (HBD) 1, 2, and 3 [16], and the cathelicidin AMP (*CAMP*) gene product LL-37 [9] were found to be among the most prominent AMPs expressed by oral cells and tissues [20]. Next, we conducted a second literature search to identify the approaches that could be applied to the transcription and expression of HNPs, HBDs, and LL-37 in oral cells and tissues. Finally, we conducted a third literature search to identify the additional functions that induced AMPs could have in regulating oral inflammation, immunity, tissue healing, and pain. The results of these three searches suggest that approaches to induce AMP transcription and expression are feasible and that induced AMP expression in the oral cavity would create an environment rich in molecules with antimicrobial activity able to regulate a variety of host defense activities and orchestrate tissue recovery events.

## 2. AMPs in the Oral Cavity

To date, between 3000 [5] and 5000 [21] natural and synthetic AMPs have been identified throughout the Animalia, Plantae, Fungi, Protista, and Monera kingdoms. These AMPs are tracked in 11 general and 9 specific databases [7]. Ramazi et al. noted that AMPs are a diverse family of peptides and have historically been grouped together based on their species of origin, similarities in their specific activities, similarities in their three-dimensional structures, similarities in their amino acid sequences, and the functions of their activities [7]. The availability of sequenced data across species can now be mined using computational methods and machine learning algorithms to identify potentially new AMPs. These algorithms can also be used to design new AMPs with novel antimicrobial activities [7].

### 2.1. AMPs Expressed in Oral Tissues

AMPs are expressed in cells and tissues of the oral cavity [22]. We searched the PubMed literature database using oral tissues, tongue, tonsils, salivary glands, gingival tissues, oral epithelium, sulcular epithelium, junctional epithelium, saliva, gingival crevicular fluid, antimicrobial peptides, defensins, cathelicidins, HBD, HNP, and LL-37 as search terms. These terms were linked in various combinations, using Boolean operators to identify AMPs reported to be expressed in oral tissues and present in oral secretions. The literature was screened for potential articles that were downloaded and read by the investigators, who selected articles that were within the search objectives for this section.

In oral tissues, HNP-1-3, HBD1-2, and S100A7 are produced by the tongue [23,24,25], HNP-4, HBD1-3, LL-37, and LEAP-1-2 are produced in the palatine tonsils [26], and HBD1-2 and LL-37 are produced by the salivary glands [23,27,28,29]. In gingival tissues, HBD1-2 are among the peptides produced by the oral epithelium, HNP-1-3 and HBD1-2 are among those produced by the sulcular epithelium, and HNP-1-3 are among those produced from the junctional epithelium [30]. LL-37, which occurs in the sulcular epithelium and junctional epithelium, is thought to be a product from neutrophils that might be present [30].

AMPs are also transcribed and expressed in deeper sites and dental pulp, as these tissues have the capacity to produce AMPs [31,32,33,34]. *DEFB1* and *DEFB4B* transcription and HBD1 and HBD2 expression were detected by RT-PCR in pulp tissue and detected by immunohistochemical staining in the cytoplasm of odontoblasts [31].

### 2.2. AMPs in Oral Secretions

Of the more than 2290 proteins detected in saliva [35,36], approximately 46 or more have reported antimicrobial activities [22,37]. Similarly, of the 100–200 proteins detected in the gingival crevicular fluid [38], seven or more are proteins have reported antimicrobial activities [39]. The physiological ranges of AMP concentrations in the saliva are high enough to support their claims as antimicrobial agents and regulators of inflammatory, immune, and healing processes (Supplemental Table S1). Of the major AMPs present, saliva contains 0.26–11.37 μM HNP-1, 0.03–5.39 μM HNP-2, 0.00–0.77 μM HNP-3, 0.00–0.04 μM HBD1, 0.00–0.03 μM HBD2, 0.00–1.36 μM HBD3, 0.56–1.11 μM LL-37 in adults and 4.45 μM LL-37 in infants [8,22,23,40,41].

## 3. Induction of Endogenous AMPs

**Scheme 1:**
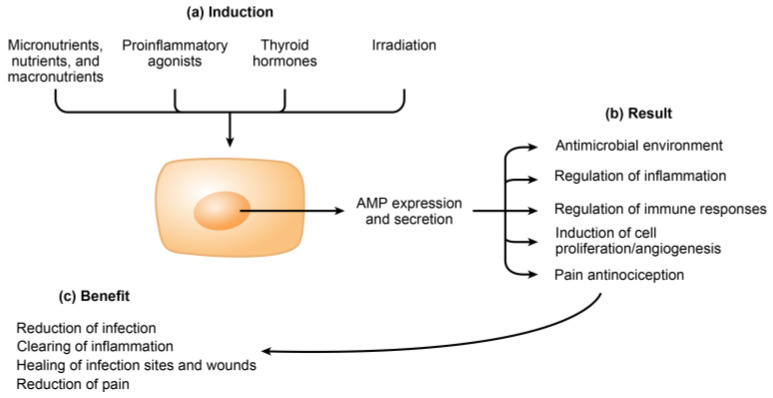
The benefits of inducing endogenous antimicrobial peptide (AMP) transcription and expression.

**Figure 1:**
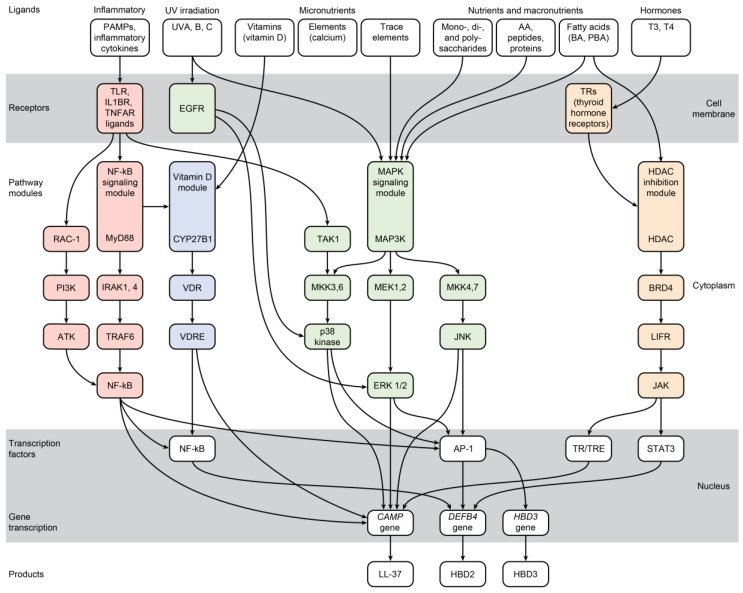
A generalized scheme showing the pathways for the induction of AMP transcription and expression via nutritional, hormonal, and physical stimuli through the MAPK signaling, vitamin D signaling, HDAC inhibition signaling, NF-kB signaling, and TR/RXT signaling modules. All signaling cascades converge onto a few transcription factors (TF) that bind to TF-specific motifs and response elements in the promoter regions of the respective AMP genes. Detailed information on each of these signaling pathways is included in Supplemental Figures S1–S4.

AMPs in cells, tissues, and salivary tissues are induced by a variety of nutritional, hormonal, or physical stimuli (Scheme 1, Table 1). We searched the PubMed literature database using micronutrients, trace elements, elements, vitamins, nutrients, macronutrients, monosaccharides, disaccharides, polysaccharides, amino acids, pyropeptides, proteins, fatty acids, proinflammatory agonists, hormones, ultraviolet (UV) C, UVB, UVA, light, near infrared radiation (NIR), antimicrobial peptides, defensins, cathelicidins, HBD, HNP, and LL-37 as search terms. These terms were also linked in various combinations using Boolean operators to identify molecules that induce AMP transcription and expression in cells, keratinocytes, oral cells, and oral tissues. Results from this search were downloaded and read by the authors. These results formed the basis for Table 1, Figure 1, Supplemental Tables S2 and S3, and Supplemental Figures S1–S4.

### 3.1. Micronutrients

#### 3.1.1. Trace Elements

Selenium [42,43,44], zinc [42,43], copper [42,43], and iron [42,43] are among the trace elements that contribute to immune competence, including maintenance of the skin barrier function, antibody production, and cellular response. Zinc is also commonly involved in signal transduction [45]. These elements are abundant in whole grains, dairy products, and seafood, and can also be consumed as supplements to boost immune function.

Experimentally, trace elements induce AMP transcription and expression (Table 1). Chickens given supplemental selenium in their diet had increased transcripts of avian β-defensins (AvBD) 6, 8, and 13 in their gastrointestinal tract and spleen [46]. Caco-2 cells treated with 20 and 50 μM zinc had increased expression of LL-37 [47]. With 20 μM zinc, LL-37 concentrations increased from 0.29 to 0.58 ng/mL in 48 h [47]. Zinc is thought to induce the phosphorylation of the extracellular signal-regulated kinase 1/2 (ERK1/2) and p38 MAP kinases [47], which regulate LL-37 expression (Supplemental Figure S1, Figure 1). Copper binding motifs built into the amino terminus of native AMPs, CM15, and citropin 1.1 presented increased antimicrobial activity against antibiotic-resistant microorganisms [48,49]. Finally, hepcidin is a cysteine-rich antimicrobial peptide that is expressed in the liver in response to iron loading and inflammation [50].

**Table 1:** Inducers of endogenous antimicrobial peptides (AMPs) to treat oral infection and inflammation.

Family of Inducers	Inducer of AMP Transcription or Expression	References
Micronutrients		
Trace elements	Selenium	[46]
	Zinc	[47]
	Copper	[48,49]
	Iron	[50]
Elements	Calcium	[51]
Vitamins	A (retinoic acid)	[52,53]
	B3 (niacin)	[54,55]
	C (ascorbic acid)	[56]
	D, D3, and calcitriol (1,25(OH)_2_D3)	[57,58,59,60,61,62,63,64,65,66,67,68]
	E (alpha-tocopherol)	[69]
Nutrients and macronutrients		
Mono-, di-, and polysaccharides	Glucose	[56,70,71]
	Lactose	[72]
	β-glucans	[73]
Amino acids, pyroglutamyl peptides, and proteins	Arginine	[74]
	Isoleucine	[74,75,76,77]
	Pyroglutamyl peptides, pyroglutamyl dipeptides, and pyroglutamyl polypeptides	[78,79]
	Bovine serum albumin (BSA)	[74]
Free fatty acids (FFA) and histone deacetylase (HDAC) inhibitors	Short FFA (≤5 carbons) including butyrate and phenylbutyrate	[15,57,72,80,81,82,83,84,85,86,87,88,89,90,91,92]
	Medium FFA (6–11 carbons) including hexanoate and heptanoate	[15]
	Long FFA (≥12 carbons) including laurate, palmitate, and oleate	[15,93]
Proinflammatory agonists		
	Pam3csk4 peptide (Toll-like receptor (TLR) 2)	[61]
	Lipopolysaccharide (LPS) (TLR4)	[23,26,94,95,96]
	CpG (TLR9)	[97]
	IL-1β	[23,98]
	TNF-α	[95,96,98]
	IFN-γ	[98]
Hormones		
	Triiodothyronine (T3)	[86]
	Thyroxine (T4)	[86]
Irradiation		
Ultraviolet	Ultraviolet C, 100–280 nm	[56]
	Ultraviolet B, 280–315 nm	[94,95,96,99,100,101,102]
	Ultraviolet A, 340–400 nm	[56]
Red light	Laser, 625 nm	[103]
Near infrared	Laser, 810 nm	[104]

#### 3.1.2. Elements

Calcium is an abundant mineral found in many food groups, including vegetables and dairy products. Calcium can be consumed as a supplement to improve skeletal, cardiovascular, and neuromuscular health and is commonly involved in cellular signaling as an enzyme cofactor or signal transducer [105].

Experimentally, calcium is known to have direct effects on innate and adaptive immune functions (Table 1). For example, when added to keratinocytes, 1.7 mM calcium can induce increased levels of *DEFB4B* and *DEFB103A* transcription and HBD2 and HBD3 expression [51]. One modeled pathway [106] featured calcium signaling through EGFR and the MAPK signaling module to ERK1/2 (Figure 1).

#### 3.1.3. Vitamins

Vitamins are micronutrients essential for normal growth and development. They are not synthesized but are required in small quantities in the diet. They form a diverse family containing vitamins A (retinoic acid), B_1_–B_12_, C (ascorbic acid), D, and E (alpha-tocopherol).

Vitamins are involved in AMP transcription and peptide expression (Table 1). Vitamin A (retinoic acid) promotes the expression of HBD2, HBD3, LL-37, and RNase7 in alveolar macrophages and respiratory epithelial cells [52],the expression of BD3 in mouse skin [53], and the expression of resistin, a small cysteine-rich AMP, in epidermal keratinocytes [107].

Little information is available on the roles of vitamins B_1_ (thiamin), B_2_ (riboflavin), B_5_ (pantothenic acid), B_6_ (pyridoxine), B_7_ (biotin), B_9_ (folic acid), or B_12_ (cyanocobalamin) in AMP transcription and peptide expression. However, vitamin B_3_ (niacin) promoted the expression of porcine β defensin (pBD) 2, protegrin 1–5, and PR39 in the gastrointestinal tract of weaned pigs, which contributed to their elimination of enterotoxigenic *Escherichia coli* K88 infections [54,55].

Vitamin C (ascorbic acid) induced *DEFB1* transcription in normal keratinocytes [56], and vitamin E (alpha-tocopherol) induced HBD1-2 expression in human gingival fibroblasts stimulated with *Porphyromonas gingivalis* lipopolysaccharide (LPS) [69].

Vitamin D has pleotropic activities, and it is important in the cellular uptake and metabolism of calcium, phosphate, and other minerals necessary for mineral metabolism and bone development [108,109]. Vitamin D regulates inflammatory events [110], prevents cancer [110], regulates endocrine system events, regulates central nervous system events, and initiates and regulates innate and adaptive immune responses [65,67]. The two main isoforms are ergocalciferol (vitamin D_2_) and cholecalciferol (vitamin D_3_) [109].

Some vitamin D_3_ comes from the dietary intake of fish, eggs, and vegetables high in vitamin D or dairy products and fruit juices fortified with vitamin D [109]. However, most vitamin D comes from the conversion of 7-dehydrocholesterol to pre-vitamin D_3_ by ultraviolet B (UVB) as a result of normal exposure to sunlight [108,109,111]. Pre-vitamin D_3_ can also come from the photochemical conversion of lumisterol and tachysterol [108,109,111]. Pre-vitamin D_3_ then becomes vitamin D_3_ and is hydroxylated in the liver to form 25(OH)D and hydroxylated in the kidneys to form 1,25(OH)_2_D3 (e.g., 1,25-dihydroxvitamin D_3_ or calcitriol) [111], while 1,25(OH)_2_D3 (calcitriol) is the physiologically active form [109].

Important to this review is the ability of vitamin D to regulate antimicrobial innate immune responses (Figure 1). This regulation occurs through the vitamin D receptor (VDR) and induces hCAP-18 gene expression [59], *CAMP* gene expression [58,66], and HBD2 gene expression [65]. The promoters of these genes contain consensus vitamin D response elements (VDRE) that mediate 1,25(OH)_2_D3-dependent gene expression [65]: 1,25(OH)_2_D3 induces antimicrobial peptide gene expression in isolated human keratinocytes [65], monocytes [65], neutrophils [65] and neutrophil progenitors [62], myeloid cells [66], EBV-transformed B-lymphocytes [62], and human cell lines [65].

Signaling (Supplemental Figure S2) occurs through the induction of pathogen recognition receptors (PRRs) (e.g., Toll-like receptor or TLR) resulting in the downstream expression of CYP27B1 (1α hydroxylase), an enzyme that converts calcifediol to 1,25(OH)_2_D3 (calcitriol) [67,112,113]. 1,25(OH)_2_D3 (calcitriol) then combines with VDR and VDRE [109]. This conversion initiates *CAMP* and *DEFB4B* transcription, resulting in LL-37 and HBD2 expression [67].

The use of micronutrients (trace elements, elements, and vitamins) to induce endogenous AMP transcription and expression is unlikely to have side effects if such micronutrients are obtained as consumable nutrients in foods. However, if consumed in excess or misused as supplements, micronutrients can induce a variety of off-target side effects. An estimated 10,176 of 32,000 (31.8%) emergency department visits per year (2004–2013) were estimated to result from adverse events related to the consumption of micronutrients [114]. Within this group, 1504 (4.7%) visits were associated with iron-induced nausea, vomiting, and abdominal pain; 1088 (3.4%) visits were associated with calcium-induced swallowing problems; and 5376 (16.8%) visits were associated with vitamin consumption. Alterations in calcium metabolism affect cellular signaling leading to changes in cell proliferation, apoptosis, autophagy, and cancer [115]. Supplements of vitamin B_6_ (>500 mg/d) can result in photosensitivity and neurotoxicity; supplements of vitamin E (800–1200 mg/d) can result in diarrhea, weakness, and blurred vision; and excess supplements of vitamin A can result in a loss of bone mineral density with an increase in fracture risk [116].

### 3.2. Nutrients and Macronutrients

Many nutrients and macronutrients can induce AMP transcription and expression [11,17]. These nutrient types include the broad categories of saccharides and polysaccharides, amino acids and proteins, and short-, medium-, and long-chain fatty acids (Table 1, Figure 1).

#### 3.2.1. Mono-, Di-, and Polysaccharides

Glucose, lactose, and complex polysaccharides such as β-glucans all induce the expression of AMPs (Table 1). Glucose induces *DEFB1* transcription in normal keratinocytes [56]. Lactose in human breast milk is thought to be involved in immune protection of the gastrointestinal tract of nursing infants by upregulating *CAMP* transcription and LL-37 expression [72]. Lactose isolated from human milk was found to induce *CAMP* transcription and LL-37 expression in the colonic epithelial cell line T84, THP-1 monocytes, and macrophages [72]. LL-37 transcription was suppressed by two different p38 antagonists, suggesting signaling via the p38 signaling pathway (Supplemental Figure S1).

β-glucans have potent immune regulatory functions [73]: they regulate the inflammatory and antimicrobial activities of neutrophils and macrophages. β-glucans are polymers of D-glucose that are linked by β-glycosidic bonds forming structural chains. These chains are found throughout the Plantae, Fungi, and Monera kingdoms as energy stores in plants and algae and as structural components in the cell walls of fungi, yeasts, and bacteria [117]; β-1,3-1,6-glucans bind a number of cellular receptors, including dectin-1, CR3, lactosylceramide, TLR2, 4, and 6, and CD36. After exposure to yeast β-D-glucans, AvBD, cathelicidin, and LEAP-2 gene expression was found to be altered in chickens [73]. Macrophages, splenocytes, and heterophils presented increased phagocytosis and bacterial killing activities. These glucans were observed to amplify humoral and cell-mediated immune responses, effectively clearing enteric infections in treated birds [73].

#### 3.2.2. Amino Acids, Pyroglutamyl Peptides, and Proteins

Amino acids regulate gene expression via the mTORC1, AMPK, and MAPK signaling pathways [11,118]. Amino acids involved in the cell signaling pathways include arginine, glutamine, glutamate, glycine, leucine, isoleucine, proline, and tryptophan. Of these, arginine, isoleucine, and several of its analogues can specifically induce epithelial HBD expression [74,75,76]. For example, when added to HCT-116 human colon cells, arginine and isoleucine can induce increased levels of *DEFB1* transcription and HBD1 expression. The administration of l-isoleucine induced a significant increase in HBD3 and HBD4, which was associated with decreased bacillary loads and tissue damage in animals infected with the *Mycobacterium tuberculosis* antibiotic-sensitive strain H37Rv and the *M. tuberculosis* strains of MDR clinical isolates, suggesting that the induction of HBDs might aid in controlling this infection [76]. Induction was transcriptional in nature (Supplemental Figure S3, Figure 1) and involved activation of the NFκB/rel family [75].

Pyroglutamyl peptides form a family of bioactive molecules [78,79] containing a free amino terminal glutaminyl group that cyclizes to form a lactam (e.g., a pyroglutaminyl group abbreviated as pyroGlu) [78]. PyroGlu peptides are common in fermented foods fish, sake, soybean paste (miso), and soy sauce (shoyu), as well as the protein lysates of corn or wheat gluten [78,79]. These peptides often remain in protein hydrolysates due to their resistance to proteinase and peptidase digestion [78]. Members include pyroGlu-Leu (pyroglutamyl leucine), pyroGlu-Tyr (pyroglutamyl-tyrosine), pyroGlu-Asn-Ile (pyroglutamyl-asparaginyl-isoleucine), and pyroGlu-Asn-Ile-Asp-Asn-Pro (pyroglutamyl-asparaginyl-isoleucyl-asparagyl-asparaginyl-proline).

PyroGlu peptides are thought to enhance the production of AMPs in the gastrointestinal tract to help maintain normal intestinal microbiota (Table 1). They regulate the colonic microbiota composition, reverse colonic dysbiosis in animals on high-fat diets, and restore colonic dysbiosis in dextran sulfate-induced colitis in mice. For example, in rats fed a high-fat diet, pyroGlu given orally increased the induction of defensin alpha 9 and rattusin, which was associated with the suppressed proliferation of Gram-positive bacteria (*Firmicutes*) [78].

Bovine serum albumin (BSA) has also been found to regulate *DEFB1* transcription and HBD1 expression [74]. BSA is a globular non-glycosylated protein isolated from bovine serum [119]. It is composed of 583 amino acids and has a mass of 66,400 Da [119]. When added to HCT-116 human colon cells, BSA induced increased levels of *DEFB1* transcription and HBD1 expression. Induced *DEFB1* transcription was related to c-myc over-expression, which suggested that *DEFB1* transcription may be regulated via c-myc signaling [74].

#### 3.2.3. Free Fatty Acids

Free fatty acids (FFAs) form a large family of carboxylic acids of saturated or unsaturated aliphatic hydrocarbon chains. FFAs are formed as breakdown products from the hydrolysis of fats and oils and readily occur in the oral cavity, in the gastrointestinal tract, and on the skin. FFAs are grouped by size into those containing five or fewer carbons (short-chain fatty acids or SCFAs), six to eleven carbons (medium chain fatty acids or MCFAs), or twelve or more carbons (long-chain fatty acids or LCFAs).

FFAs have very potent innate immune properties, including direct antimicrobial activities against viruses, bacteria, and fungi [120] and the ability to induce AMP transcription and expression (Table 1). Generally, FFAs shorter than four carbons or longer than seven carbons have only a marginal ability to induce *CAMP* transcription and LL-37 expression [15]. In laboratory studies, SCFAs including butyrate, phenylbutyrate, glyceryl tributyrate, benzyl butyrate, and valerate were found to be potent inducers of LL-37 [15,57,72,80,82,83,84,85,86,87]; MCFAs including hexanoate (six carbons) and heptanoate (seven carbons) are moderate inducers of LL-37 [15]; and LCFAs including laurate, palmitate, and oleate are marginal inducers of LL-37 [15] but moderate inducers of HBD2 expression [93]. Sodium butyrate upregulates LL-37 gene expression in PNEC cells and NCI-H292 cells [80] and induces pBD2, pBD3, epididymis protein 2 splicing variant C (pEP2C), and protegrin expression in porcine IPEC-J2 intestinal epithelial cells, 3D4/31 macrophages, and primary monocytes [83]. Phenylbutyrate increases *CAMP* transcription in VA10, HT-29, A498, and U937 cell lines [87]. *DEFB1* transcription was increased in the lung epithelial cell line VA10, but decreased in the monocytic cell line U937 [87]. Glyceryl tributyrate, benzyl butyrate, and 4-phenylbutyrate were comparable to butyrate in activity [83]. Valerate induced *CAMP* gene expression in HT-29 and U937 cell lines [15]. Hexanoate and heptanoate induced LL-37 gene expression in HT-29 and U937 cell lines [15] and laurate, palmitate, and oleate induced expression of HBD2 in human sebocytes [93]. In clinical trials, sodium butyrate provided protection for individuals infected with shigellosis [85].

The induction of AMP transcription and expression by FFAs occurs through multiple pathways (Supplemental Figures S1–S3 and Figure 1). Phenylbutyrate and its analogue alpha-methylhydrocinnamate induce *CAMP* transcription through the ERK1/2 and c-Jun N-terminal kinase (JNK) signaling pathways [87]. Butyrate and valproate also act as histone deacetylase (HDAC) inhibitors (Supplemental Figure S4, Figure 1). HDAC is an enzyme that removes the acetyl group from DNA histones, making it less accessible to transcription factors. HDAC regulates a number of important cellular proteins, including histones h3 and h4, MMP9, and others, including AMPs. The inhibition of this enzymatic process by butyrate and valproate likely makes DNA more accessible to transcription factors, thus increasing *DEFB4B* transcription (Supplemental Figure S4).

#### 3.2.4. Foodstuffs

Foodstuffs contain complex macronutrients that can induce AMP transcription in oral cells, suggesting that their consumption contributes to the presence of AMPs in the oral cavity (see Section 2.1 and Section 2.2 above). For example, human milk oligosaccharide 3-fucosyllactose with and without 2’-fucosyllactose induced *DEFB4B* transcription in human gingival cells, suggesting that these oligosaccharides may stimulate the oral mucosal expression of HBD2 without inducing a proinflammatory response [121]. Avocado extracts contain natural sugars that induce *DEFB4B* and *DEFB103B* transcription in keratinocytes [122,123]. Fruit extracts containing gallic acid induce the expression of HBD2 in primary human gingival epithelial cells [124].

Green tea extracts containing polyphenols induce the expression of pBD1 and pBD2 in porcine jejunal epithelial cells IPEC-J2 [125] and *DEFB1*, as well as *DEFB4B* expression in gingival epithelial cells [126]. Black tea extracts containing theaflavins induce HBD1, 2, and 4 expression in oral epithelial cells [127]. Peonies are a component of herbal medicine, and their extracts containing paeoniflorin upregulate HBD2 expression in human bronchial epithelial cells through the p38 MAPK, ERK1/2, and NF-κB signaling pathways [128].

Finally, foodstuffs containing complex macronutrients that can induce AMP transcription have been proposed to protect the overall general health of the oral cavity and gastrointestinal tract. In a unique synergy, flours from amaranth, millet, soybean, and sesame grains increased *DEFB4B* transcription in human HFK cells co-stimulated with *E. coli* [12].

Overall, the consumption of nutrients, macronutrients, and foodstuffs is thought to be generally safe [116]. The use of nutrients, macronutrients (mono-, di-, and polysaccharides, amino acids, pyropeptides, proteins, and fatty acids), and foodstuffs to induce endogenous AMP transcription and expression would be beneficial and suitable for long-term health protection. The specialized metabolites or concentrated extracts that are formulated as dosage forms would provide therapeutic consideration. However, a few can induce a variety of off-target side effects if consumed in excess. Arginine (>6 g/day), for example, can induce nausea and diarrhea [129], and excess consumption of protein powders can result in ketosis [116].

### 3.3. Proinflammatory Agonists

These agonists are well-known and potent inducers of proinflammatory and AMP responses in a variety of cells and tissues (Supplemental Figure S3, Figure 1). They involve a vast number of microbial antigens and products with unique pathogen-associated molecular patterns (PAMPs), as well as host-derived proinflammatory cytokines released from damaged, stressed, or abnormal cells and tissues. To recognize and respond to the presence of these molecules, host cells contain both surface PRRs and cytokine receptors. This process also occurs in oral tissues [130]. These PRRs encompass the TLR family, Nod-like receptor/nucleotide-binding oligomerization domain-like receptor (NLR), retinoic acid-inducible gene I-like receptor (RLR), C-type lectin receptor (CLR), and AIM2-like receptor (ALR) groups. PRRs that initiate AMP transcription include TLR2-6 and 9 [131].

Lipoproteins, lipoteichoic acid, triacyl bacterial lipopeptides, and fungal zymosans are TLR2 and TLR6 ligands [61]; double stranded viral RNA is a TLR3 ligand; and bacterial LPS [23,26,95] and *P. gingivalis* recombinant hemagglutinin B (rHagB) [132] are TLR4 ligands. LPS induces *DEFB1*, *DEFB4B*, and *CAMP* transcription and HBD1, HBD2, and LL-37 expression in a variety of cell types [95,96]. Unmethylated DNA CpG is a TLR9 ligand.

Proinflammatory cytokines including IL-1β and TNF-α bind and signal via IL1R [133], TNFR-1, and TNFR-2 [134]. ILR1 belongs to the Toll-IL1-receptor (TIR) superfamily, and TNFRs belong to the TNF receptor superfamily. IL-1β and TNF-α induce *DEFB1*, *DEFB4B*, and *CAMP* transcription and HBD1, HBD2, and LL-37 expression in a variety of cell types [95,96]. Agonist–receptor engagement then results in the initiation of specific cascades through the MAPK [131] and NF-κB [131] signaling modules (Supplemental Figure S3, Figure 1).

The use of proinflammatory agonists such as LPS to induce endogenous AMP transcription and expression is unlikely to be a popular or feasible approach. Systematic LPS administration can systemically induce inflammation, proinflammatory cytokine production, fever, and septic shock [135]. LPS present in food and food supplements, however, seems to be tolerated [135].

### 3.4. Thyroid Hormones

Thyroid hormones regulate metabolism and immune functions [136], and an excess or deficiency of these hormones can have profound effects in the oral cavity [137]. It is possible that these hormones may also induce the transcription and expression of AMPs in the oral cavity (Table 1). This activity is supported by a report showing that colonic epithelial cells treated with thyroid hormones triiodothyronine (T3) and thyroxine (T4) induced *CAMP* transcription [86]. LL-37 expression was induced by both T3 (2.5 nM–1.0 μM for 3–30 h) and T4 (2.5–10 nM for 24 h) [86].

There are a number of possible mechanisms for this phenomenon, one of which is receptor mediated. T3 and T4 bind to cellular thyroid hormone receptors (TRs), which are ligand-activated transcription factors (TFs). TRs then bind to thyroid hormone response elements (TREs) together with retinoid X receptors (RXR) in the promotor regions of their target genes to regulate gene expression. A good example is the presence of TR/TRE binding sites in the promoter region of the *CAMP* gene [86]. Another mechanism may repress HDACs similar to butyrate and phenylbutyrate (Supplemental Figure S4). Finally, RXR may also be involved with VDR and VDRE in vitamin D-induced *CAMP* and *DEFB4B* transcription and LL-37 and HBD2 expression [138].

While thyroid hormones are associated with changes in the oral cavity, altering thyroid hormone concentrations to induce endogenous AMP transcription and expression would not likely be a valid approach. Any advantages would be offset by numerous and more severe consequences that could be associated with metabolic complications [137].

### 3.5. Irradiation

AMP transcription and expression is induced in cells and tissues after exposure to radiation within select ranges in the electromagnetic spectrum [139,140,141]. Irradiation induces AMP expression in normal human skin and regulates AMP expression in the skin of individuals with dermal diseases (Table 1 and Table 2). It also has the potential to induce AMP expression in oral tissues for treatment of oral infections and inflamed areas to enhance healing processes [18,142].

#### 3.5.1. UVC

The use of UVC irradiation is relatively new compared to that of other areas in the electromagnetic spectrum. UVC irradiation (100–280 nm) alone is directly antimicrobial and rapidly kills microorganisms but is not toxic to oral cells [143,144]. Irradiation also induces the transcription and expression of chemokines, cytokines, and growth factors that are beneficial to endodontic tissue regeneration [143]. In exposed cells, UVC induces AMP expression. For example, Antonio Cruz Diaz et al. found that UVC exposure of human keratinocytes for 5 and 10 min induced *CAMP* and *DEFB1* transcription [56].

UVC is thought to signal through the p38 MAPK, ERK1/2, and JNK pathways [18]. Abbreviated pathways are shown in Supplemental Figure S1 and Figure 1. UVC stimulates EGFR signaling to p38 MAPK. UVC also activates EGFR and signals through SRC > RAS > raf > MAP2K1 to ERK1/2. UVC can also activate PKC and MAP2K1 to ERK1/2. ERK1/2 then activates transcription factor AP-1. Alternately, UVC can activate sphingomyelinase, which activates JNK. Activated (phosphorylated) JNK in turn signals to AP-1.

#### 3.5.2. UVB

UVB irradiation (280-315 nm) alone is directly antimicrobial but also induces cells and tissues to express AMPs (Supplemental Table S2). In cultured cells, irradiation induces *DEFB4B*, *DEFB103A*, *RNASE7*, *S100A7*, and *CAMP* transcription and HBD2, HBD3, RNase7, S100A7, and hCAP18/LL-37 expression [100,102,104]. For example, keratinocytes from neonatal foreskin and human keratinocyte HaCat cells treated with UVB irradiation (30 or 100 mJ/cm^2^) induced *DEFB1* and *DEFB4B* transcription, whereas transformed human keratinocyte A431 cells did not [96]. In tissue biopsies, UVB irradiation increased the expression of HBD and LL-37 [95]; upregulated skin chemerin [99], a potent chemoattractant with antimicrobial activity [145]; and induced levels of *CAMP* transcription [102].

In clinical studies, UBV irradiation induces AMP expression in normal human skin and regulates AMP expression in the skin of individuals with atopic eczema, psoriasis, dermatitis, and vitiligo [141]. UVB increased hCAP18 and vitamin D receptor expression in healthy skin biopsies at 24 h posttreatment [102]. In patients with atopic eczema, HBD1 expression was decreased, and HBD2 expression was increased, in the epidermis, which is thought to lead to the recurrent skin infections seen in this condition [146]. After treatment of atopic eczema skin with NB-UVB phototherapy, values returned to normal. HBD1 expression was increased, and HBD2 expression was decreased, compared to responses in healthy controls [146].

UVB is thought to signal through the p38 MAPK, ERK1/2, and JNK pathways [18]. Abbreviated pathways are shown in Supplemental Figure S1 and Figure 1. UVB can stimulate the phosphorylation of p38 MAPK or stimulate EGFR signaling to p38 MAPK. UVB activates ERK1/2 in multiple pathway combinations: PKC > ERK1/2; PCK > JNK > ERK1/2; PI3K > PKC > ERK1/2; and/or PI3K > PKC > JNK > ERK1/2. Activated ERK1/2 then signals to AP-1. UVB activates JNK in multiple pathway combinations: UVB activates PKC > JNK and/or PI3K > PKC > JNK. Activated (phosphorylated) JNK, in turn, signals to AP-1.

#### 3.5.3. UVA

UVA (340–400 nm) is a longer wavelength that also regulates AMP transcription and expression in keratinocytes. In human keratinocytes, Cruz Diaz et al. found that 5 min of UVA irradiation induced *DEFB1* transcription, whereas 10 and 20 min of UVA irradiation reduced *DEFB1* transcription [56].

Clinically, UVA can regulate AMP transcription, which is correlated with clinical improvement of localized scleroderma [147]. Prior to treatment, *DEFB1*, *DEFB4B*, and *DEFB103A* transcription levels were higher in localized scleroderma compared to normal skin. After treatment with UVA, clinical scores and areas of treatment improved. *DEFB1* transcription decreased in localized scleroderma compared to normal skin. *DEFB103A* transcription decreased in localized scleroderma but increased in normal skin [147].

UVA signals through the p38 MAPK, ERK1/2, and JNK pathways [18]. Abbreviated pathways are shown in Supplemental Figure S1 and Figure 1. UVA can phosphorylate p38 MAPK. Activated p38 MAPK then signals to STAT1. UVA can activate EGFR and signal to ERK1/2. UVA can also signal to PLC with Ca^2+^ to PRKCA through RAS to ERK1/2. ERK1/2 then signals through RPS6KA5 to STAT1. Finally, UVA can activate JNK directly. Activated (phosphorylated) JNK then signals to AP-1 and STAT1.

#### 3.5.4. Red Light

The red-light region occurs within the visible spectrum at 625–740 nm. Lasers emitting light at 625 nm can induce *CAMP* and *DEFB1* transcription and LL-37 expression in immortalized gingival fibroblasts infected with *P. gingivalis* [103].

#### 3.5.5. Near-Infrared Irradiation (NIR)

NIR occurs just outside the visible spectrum at 800 to 2500 nm. NIR at 810 nm induces higher *DEFB4B* transcription in oral fibroblast cells than in oral keratinocytes [104]. Transcription was higher at lower fluences, and the induction of HBD2 occurred via activation of the TGF-B1 pathway and signaling through the Smad and non-Smad pathways [104].

Using irradiation to induce AMP transcription and expression would be an attractive approach. Irradiation can be strictly controlled in the clinic and used locally in the oral cavity. This measure can avoid systemic or long-term over exposure. The dental community has been using UV irradiation in the oral cavity for over 50 years to whiten teeth and polymerize compounds used in restorations and fillings [142]. Since the 1980s, narrow-band UVB (311–312 nm) has been used successfully to reduce the erythema response in psoriasis patients, and UVA (340–400 nm) has been used to treat atopic dermatitis [141]. However, safety can be compromised if exposure doses exceed therapeutic levels.

### 3.6. Synergy among Inducers

Increases in AMP transcription and expression are induced in cells after exposure to multiple AMP inducers (Supplemental Table S3). Co-exposure to lactose and butyrate or phenylbutyrate [86], butyrate and cAMP [82], phenylbutyrate and vitamin D_3_ [87], and vitamin D_3_ and LPS [65] were all found to induce higher AMP expression levels. In a well-studied example of synergy among lactose and phenylbutyrate, the regulatory pathways were assessed in their induction of the *CAMP* gene [86]. The colonic epithelial cell line HT-29 was treated with lactose, phenylbutyrate, and lactose + phenylbutyrate. Induced proteins assessed via mass spectroscopy and pathway analysis showed no additional metabolic pathways detectable in colonic cells treated with lactose + phenylbutyrate compared to proteins/pathways activated in cells treated with lactose or phenyl butyrate separately. Metabolic pathways activated with lactose included glycolysis and the pentose phosphate pathway, and the metabolic pathways activated with phenylbutyrate included those associated with the biosynthesis of steroids and butyrate/propionate metabolism [86].

Synergy also occurs among proinflammatory cytokines [98]. Various combinations of IL-1β, TNF-α, and IFN-γ induced *DEFB4B* and *DEFB103A* transcription. Depending on the combination of proinflammatory cytokines and HBD examined, *DEFB4B* and *DEFB103A* transcription can vary from a 4- to 150-fold increase.

## 4. Roles of AMPs in Inflammation, Immunity, Healing, and Pain

Induced HNPs, HBDs, and LL-37 have additional roles beyond their antimicrobial activities. Their local presence can regulate the intensity of inflammation; regulate innate and adaptive immune functions, and accelerate angiogenesis, vasculogenesis, and wound healing activities. We searched the PubMed literature database using innate immunity, chemotaxis, cell migration, proliferation, cytokine response, gene expression, microbial antigens, pathway signaling, adaptive immunity, antibody response, Th1, Th2, wound healing, angiogenesis, cytotoxicity, apoptosis, and pain together with antimicrobial peptides, defensins, cathelicidins, HBD, HNP, and LL-37 as search terms. These terms were linked in various combinations using Boolean operators to identify AMPs reported to also have roles in inflammation, immunity, healing, and pain. Appropriate articles were downloaded and read by the authors. The information from this search was used to construct Supplemental Table S1 and Table 2.

Although there are wide ranges in the concentrations of AMPs used in many studies (Supplemental Table S1, Table 2), generally AMP-induced activities are concentration-dependent [148]. At lower concentrations (e.g., 0.01 to 0.22 μM), AMPs have chemotactic activities, bind microbial antigens, enhance cytokine responses, promote cell migration, initiate angiogenesis, enhance antibody responses, attenuate cytokine responses, induce cell proliferation, suppress apoptosis, promote cell migration, activate pathway signaling, and enhance wound closure (Table 2). At intermediate concentrations (e.g., 1.00 to 2.54 μM), AMPs attenuate signaling pathways, begin to induce cell cytotoxicity, enhance gene expression, and decrease cell numbers in experimental assays (Table 2). At higher concentrations (e.g., 4.45 to 58.00 μM), AMPs attenuate the expression of genes, increase the production of cell markers, induce Th1 cytokine profiles, delay wound closure, and enhance wound healing (Table 2). For example, at lower concentrations, defensins do not induce TNF-α or IL-1β expression in monocytes or macrophages [149,150], but at higher concentrations, defensins induce chemokine and cytokine production in epithelial cells, keratinocytes, monocytes, and macrophages [151,152,153]. LL-37 induces CXCL8 in epithelial cells and macrophages [154]. At their highest concentrations, AMPs are cytotoxic for a variety of cells.

### 4.1. Roles of AMPs in Inflammation

AMPs regulate inflammatory processes in part by (i) altering proinflammatory agonist binding to cells and (ii) altering intracellular cytokine signaling pathways. Regulation occurs in both directions. In some circumstances, AMPs increase the expression of proinflammatory cytokines in stimulated cells [152,155,156,157], but in other circumstances, AMPs decrease the expression of proinflammatory cytokines [158,159].

AMPs can directly bind to microbial agonists [160,161,162,163,164,165], which likely prevents their binding to cells. AMPs can also alter the binding of microbial agonists to cells [166,167,168]. HNP-1,2 and HBD3 bind to *P. gingivalis* recombinant fimbrillin A (rFimA) [160]; HNP-1,2 and HBD3 bind to *P. gingivalis* rHagB [160,163]; and LL-37 binds to LPS [169]. The binding of AMPs to microbial agonists attenuates proinflammatory cytokine responses. HNP-1-3 attenuates the expression of proinflammatory cytokines from macrophages [170]. HNP-1 attenuates the expression of IL-1β, but not TNF-α, from LPS-treated monocytes [171]. Histatin 5, HBD1, and HBD3 attenuate the expression of IL6, IL10, GM-CSF, and TNF-α from rHagB-treated dendritic cells [163,164], and LL-37 attenuates the expression of proinflammatory cytokines to TLR2, 4, and 9 agonists [172,173].

AMPs alter the intracellular signaling pathways. As an example, HBD3 rapidly enters cells and once in the cytoplasm, prevents the expression of genes related to the activation of proinflammatory responses to LPS/KDO_2_-Lipid A [158]. The inhibitory effects likely occur downstream of TLR4 activation by LPS. Furthermore, HBD3 dramatically reduced the number of genes expressed in KDO_2_-lipid A-treated macrophages [158]. LL-37-treated PBMC, CD14^+^ monocytes, dendritic cells, B-lymphocytes, and T-lymphocytes all produced different profiles of intracellular cytokines [174]. Scott et al. showed that LL-37 increased the expression of 29 genes and decreased the expression of 20 genes encoding chemokines and chemokine receptors [154]. Additionally, Mookherjee et al. showed that LL-37 increased the expression of 475 genes in stimulated CD14^+^ monocytes [174]. LL-37 activated the p38, ERK1/2, and JNK; IκBα/NFκB; and PI3K signaling pathways and the AP-1, AP-2, E2F1, EGR, NFκB, and SP-1 transcription factors [174].

### 4.2. Roles of AMPs in Immunity

Once expressed, AMPs regulate important innate and adaptive immune-related activities [9,175,176,177,178]. In innate immunity, AMPs induce chemotactic activities, induce cell migration, increase the production of cellular markers, induce cell proliferation, suppress apoptosis, and increase cellular cytotoxicity (Table 2).

In adaptive immunity, AMPs induce adaptive immune responses, enhance antibody responses, and increase cell survival (Table 2). LL-37 (11.12 μM) was found to induce dendritic cell differentiation, increase FITC-labeled dextran antigen uptake, increase co-stimulatory molecule CD11b, CD86, and CD83 expression, and enhance the Th1 cytokine response [179]. Dendritic cells exposed to LPS produced a T helper type 1 (TH1) cell inducing cytokine profile [179].

### 4.3. Roles of AMPs in Angiogenesis, Vasculogenesis, and Wound Healing

AMPs clearly accelerate wound healing events [178]. AMPs chemoattract a variety of cells important for wound healing. They increase cell migration, increase angiogenesis, increase vasculogenesis, and facilitate wound closure (Table 2). LL-37 attracts fibroblasts, microvascular endothelial cells, and human umbilical vein endothelial cells [180]. AMPs can also increase cell proliferation [152,181], which facilitates the closure of cell monolayers in scratch models. LL-37 increases fibroblast proliferation, induces human microvascular endothelial cell and human umbilical vein endothelial cell proliferation, and stimulates reepithelialization [180,182,183].

At concentrations of 0.3–5.9 μM, AMPs participate in cell proliferation and wound repair activities. These activities include a large variety of cells involved in the repair of injured tissues (epithelial cells, PLE epithelial cells, corneal epithelial cells, and fibroblasts), cells involved in allergic inflammation (mast cells and eosinophils), cells involved in angiogenesis (endothelial cells), and some cancer cell lines (A549 carcinoma cells, NCI tumor cells, and human epithelial carcinoma KB cell line).

### 4.4. Roles of AMPs in Pain Nociception

A new and exciting area of AMP functions is the potential of AMPs to be antinociceptive. While inducers of endogenous AMP expression are not yet known to be directly antinociceptive, it is tempting to speculate that they may have stimulatory activities on the MAPK (p38, JNK, and ERK1/2) and NF-κB signaling pathways that produce AMPs and cytokines involved in pain reduction [18]. Blockage of the peripheral activation of ERK1/2 was thought to be antinociceptive on melittin-induced persistent spontaneous nociception and hyperalgesia [184].

Recent work in a variety of fields has demonstrated that small peptides with antimicrobial activities are also involved in nociceptive and antinociceptive responses. These peptides come from insects [184,185,186,187], marine organisms and fish [188,189], cationic arginine-rich peptides [190], lanthipeptides [191], and endocrine cells [192]. Many peptides have been reported to have stimulatory or inhibitory activities on the NF-κB [185] and MAPK (ERK1/2, JNK and p38) [184] signaling pathways. These findings could form the basis to assess the roles of HNPs, HBDs, and LL-37 in pain reduction.

## 5. Potential Applications of Inducing Endogenous AMPs

The use of a specific inducer would depend upon the overall objective [6,10,12,13,14,15,16,17,18]. AMPs could be induced as prophylactic or preventative measures to bolster innate resistance for improved health or preventing infection [193]. An enhanced antimicrobial barrier would have all the advantages of an alert innate immune system on standby in individuals prone to oral infections including stomatitis and denture stomatitis or about to undergo a surgical procedure. Alternately, AMPs could be induced to treat an emerging or established oral infection. Expressed AMPs would also be available to control acute inflammation and pain antinociception. The presence of AMPs would have the added advantage of inducing additional innate and adaptive immune responses and then facilitating or expediting local wound healing, tissue regeneration, and regrowth.

Inducing the expression of AMPs through micronutrients, nutrients, and macronutrients (Table 1) would be most applicable to improving general health within the oral cavity and gastrointestinal tract. This nutrient-based approach could be used to deliver dietary supplements to maintain normal animal and human growth and health. The actions of the supplements would occur in the epithelium lining, oral cavity, and gastrointestinal tract. Doses would not be readily known, and treatments would generally not be toxic.

Inducing the expression of AMPs by irradiation would be primarily applicable to clearing focal infections. Treatments would likely be highly focused and controlled in a clinical setting. Expressed AMPs would have direct antimicrobial activities and regulate local defenses to attenuate inflammation, ameliorate pain, and help oral tissues heal. Applications include controlling infections in the oral cavity and include root canals. The wavelength and dose of UV irradiation can be easily controlled to facilitate healing and tissue regeneration, and treatments would not be toxic at therapeutic levels.

Dental procedures often have accompanying pain. Treating dental infections, as well as reducing the associated pain, would have popular benefits. However, the ability of AMPs to be antinociceptive is not yet fully known and remains under investigation.

## Figures and Tables

**Table 2:** Roles of AMPs in inflammation, immunity, healing, and pain. The activities below were compiled from results reported in Supplemental Table S1.

AMP Concentration (μM)	Activity	AMPs Involved (Reported μM Concentrations)
Roles of AMPs in inflammation		
0.02–0.30	Binds microbial antigens	LL-37 (0.02 μM), HBD3 (0.19 μM), HBD1 (0.25 μM), and Histatin 5 (0.30 μM)
1.94	Attenuated pathway signaling	HBD3 (1.94 μM)
4.45	Attenuated gene expression	LL-37 (4.45 μM)
0.19–2.50	Attenuated cytokine response	HBD3 (0.19 μM), LL-37 (0.22–2.50 μM), HBD1 (0.25 μM), and HNP-1,2 (0.29 μM)
1.11	Activated pathway signaling	LL-37 (1.11 μM)
2.32	Enhanced gene expression	HNP-1-3 (2.32 μM)
0.03–29.00	Enhanced cytokine response	HBD1 (0.03–5.08 μM), LL-37 (0.11–11.12 μM), HBD2 (0.46–4.61 μM), HNP-1-3 (0.87–29.0 μM), and HBD3 (0.97–3.87 μM)
Roles of AMPs in immunity		
0.01–29.00	Chemotactic activity	HNP-1-3 (0.01–29.00 μM) and LL-37 (0.22-4.45 μM)
1.00–2.32	Promoted cell migration	LL-37 (1.00 μM) and HNP-1-3 (2.32 μM)
11.12	Increased cell markers	LL-37 (11.12 μM)
0.22-11.12	Induced proliferation	LL-37 (0.22–2.22 μM), HNP-1-3 (0.29–2.90 μM), HBD3 (0.97–1.55 μM), HBD2 (1.15–2.31 μM), and HBD1 (1.27 μM)
	Induced Th1 cytokine profile	LL-37 (11.12 μM)
0.19–1.45	Enhanced antibody response	HBD3 (0.19 μM), HBD2 (0.23 μM), HBD1 (0.25 μM), and HNP-1-3 (0.29–1.45 μM)
1.00	Suppressed apoptosis	LL-37 (1.0 μM)
2.54–12.70	Decreased cell numbers	HBD1 (2.54–12.70 μM)
2.22–14.50	Cell cytotoxicity	LL-37 (2.2–11.12 μM), HBD2 (6.92 μM), HNP-1-3 (14.50 μM)
Roles of AMPs in angiogenesis, vasculogenesis, and wound healing		
0.12–58.00	Angiogenesis	HBD2 (0.12 μM) and HBD3 (58.00 μM)
0.11–2.31	Promoted cell migration	HBD3 (0.11–0.97 μM), HBD2 (0.12–2.31 μM), and HBD4 (2.22 μM)
1.16–58.00	Enhanced wound closure	LL-37 (0.11–2.50 μM), HBD2 (0.12 μM), HNP-1-3 (1.16–2.32 μM), and HBD3 (58.00 μM)
14.50	Delayed wound closure	HNP-1-3 (14.50 μM)
58.00	Enhanced wound healing	HBD3 (58.00 μM)
Roles of AMPs in pain nociception		
0.2–6.0 mg/kg	Pain antinociception	Alloferon (0.10 μM), PCD-1 (3.69 μM), Ueq 12-1 (0.2 mg/kg), CgA (0.5 mg/kg), and AMV (6 mg/kg)

## Data Availability

Not applicable.

## Supplementary Materials

The following supporting information can be downloaded at https://www.mdpi.com/article/10.3390/antibiotics12020361/s1. Supplemental Figure S1. MAPK-dependent induction of antimicrobial peptides (AMPs) [194,195,196]; Supplemental Figure S2. Vitamin D induction of antimicrobial peptides (AMPs); Supplemental Figure S3. NF-κB-dependent induction of antimicrobial peptides (AMPs) [195,196,197,198]; Supplemental Figure S4. Butyrate (BA) and phenylbutyrate (PBA) inhibition of histone deacetylase (HDAC) [199,200,201]; Supplemental Table S1. Many of the roles antimicrobial peptides (AMPs) play in inflammation: innate and adaptive immunity, angiogenesis, vasculogenesis, wound healing, and pain antinociception [202,203,204,205,206,207,208,209,210,211,212,213,214,215,216,217,218,219,220]; Supplemental Table S2. Irradiation induction of antimicrobial peptides (AMPs); and Supplemental Table S3. Synergistic activity of molecules on induction of antimicrobial peptides (AMPs) [221].
