# Supplementary material for: Induction of Endogenous Antimicrobial Peptides to Prevent or Treat Oral Infection and Inflammation
Source: Antibiotics (Basel). 2023 Feb 9;12(2):361. doi: 10.3390/antibiotics12020361 (PMC9952314; doi:10.3390/antibiotics12020361)

antibiotics

Supplemental Fig S1. MAPK-dependent induction of antimicrobial peptides (AMPs). A scheme showing pathways leading to MAPK-dependent induction of AMP transcription and expression by micronutrients (zinc), elements (calcium), nutrients and macronutrients (isoleucine, lactose, β-glucans, butyrate (BA), and phenylbutyrate (PBA)), and ultraviolet (UV) C, B, and A irradiation. Pathways leading to induction of AMP transcription and LL-37 and HBD2 expression were compiled from results reported in studies by a) Talukder et al.. 2011 [47], b) Cederlund et al. 2013 [72], c). Krisanaprakornkit et al. 2002 [194], d) Nishimura et al. 2003 [12], e) Steinmann et al. 2009 [87], f) Li et al. 2009 [195], g) Schauber et al. 2003 [89], h) Schwab et al. 2007 [90], i) Morio et al. 2022 [18], j) KEGG calcium signaling pathway [106], and k) Konno et al. [77], and l) Steubesand et al. 2009 [196].

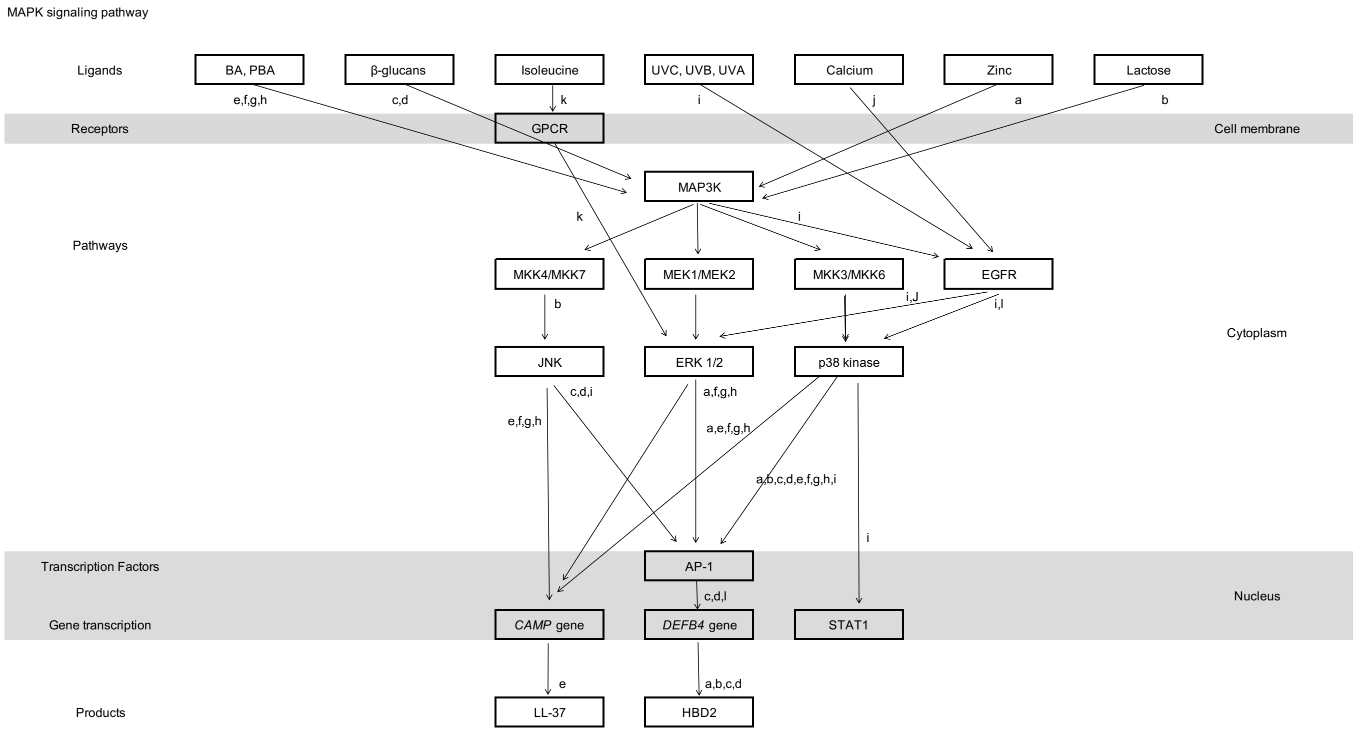

Supplement: Supplementary file 1 [file antibiotics-12-00361-s001.zip › Antibiotics Suppl Fig S1 (02-06-23).pdf]
