# Supplementary material for: Induction of Endogenous Antimicrobial Peptides to Prevent or Treat Oral Infection and Inflammation
Source: Antibiotics (Basel). 2023 Feb 9;12(2):361. doi: 10.3390/antibiotics12020361 (PMC9952314; doi:10.3390/antibiotics12020361)
Supplement: Supplementary file 1 [file antibiotics-12-00361-s001.zip › Antibiotics Suppl Fig S2 (02-06-23).pdf]

antibiotics

1

Supplemental Fig S2. Vitamin D induction of antimicrobial peptides (AMPs). A scheme showing pathways leading to vitamin D induced AMP transcription and expression. Proinflammatory ligands engage Toll-like receptor (TLR) 2. TLR signaling activates the NF-kB module resulting in the expression of CYP27B1 (1α hydroxylase), an enzyme that converts calcifediol (vitamin D) to calcitriol (1,25(OH)$_2$D3). This enzymatic process is thought to occur locally in response to infection [67]. Calcitriol combines with the vitamin D receptor (VDR) and vitamin D response element (VDRE). The promoter region of *CAMP* gene contains VDRE binding motifs that result in *CAMP* transcription and LL-37 expression [13]. Secondary to this pathway is *DEFB4B* transcription and HBD2 expression resulting from the TLR2-activated NF-kB module. The promoter region of the *DEFB4B* gene contains several NF-kB binding motifs that likely leads to *DEFB4B* transcription and HBD2 expression [13]. Pathway connections leading vitamin D induction of AMP transcription and LL-37 and HBD2 expression were based off of studies by a) White, 2022 [67], b) Campbell et al. 2012 [13], c) Edfeldt et al. 2010 [113], d) Liu et al. 2006 [112], e) Wang et al. 2004 [65], and f) Misawa et al. 2009 [60].

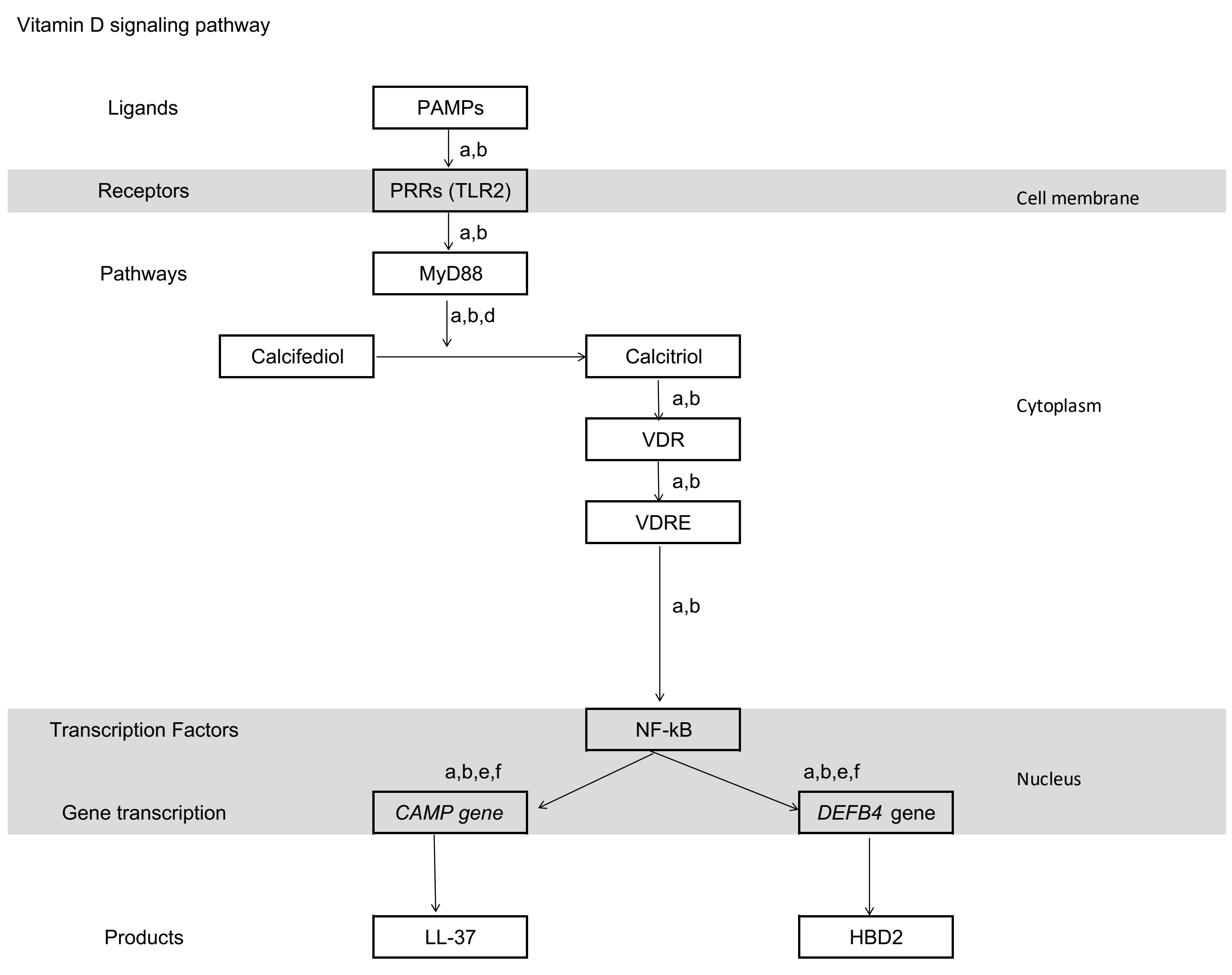

2
