# Supplementary material for: Induction of Endogenous Antimicrobial Peptides to Prevent or Treat Oral Infection and Inflammation
Source: Antibiotics (Basel). 2023 Feb 9;12(2):361. doi: 10.3390/antibiotics12020361 (PMC9952314; doi:10.3390/antibiotics12020361)

antibiotics

Supplemental Fig S3. NF-κB-dependent induction of antimicrobial peptides (AMPs). A scheme showing pathways leading to NF-κB-dependent induction of AMP transcription and expression by proinflammatory cytokines (IL-1β and TNF-α), nutrients and macronutrients (I-isoleucine, BA, and PBA), proinflammatory agonists (pathogen-associated molecular patterns or PAMPs and microbial cells). Pathways leading to induction of *AMP* transcription and LL-37 and HBD2 expression were based off of studies by a) Froy 2005 [131], b) Nishimura et al. 2003 [12], c) Mookherjee et al. 2006 [172], d) Li et al. 2009 [195], e) Wehkamp et al. 2006 [197], f) Chen et al. 2020 [17], g) Steubesand et al. 2009 [196], h) Nakatsuji et al. 2010 [93], and i) Drover et al. 2008 [198].

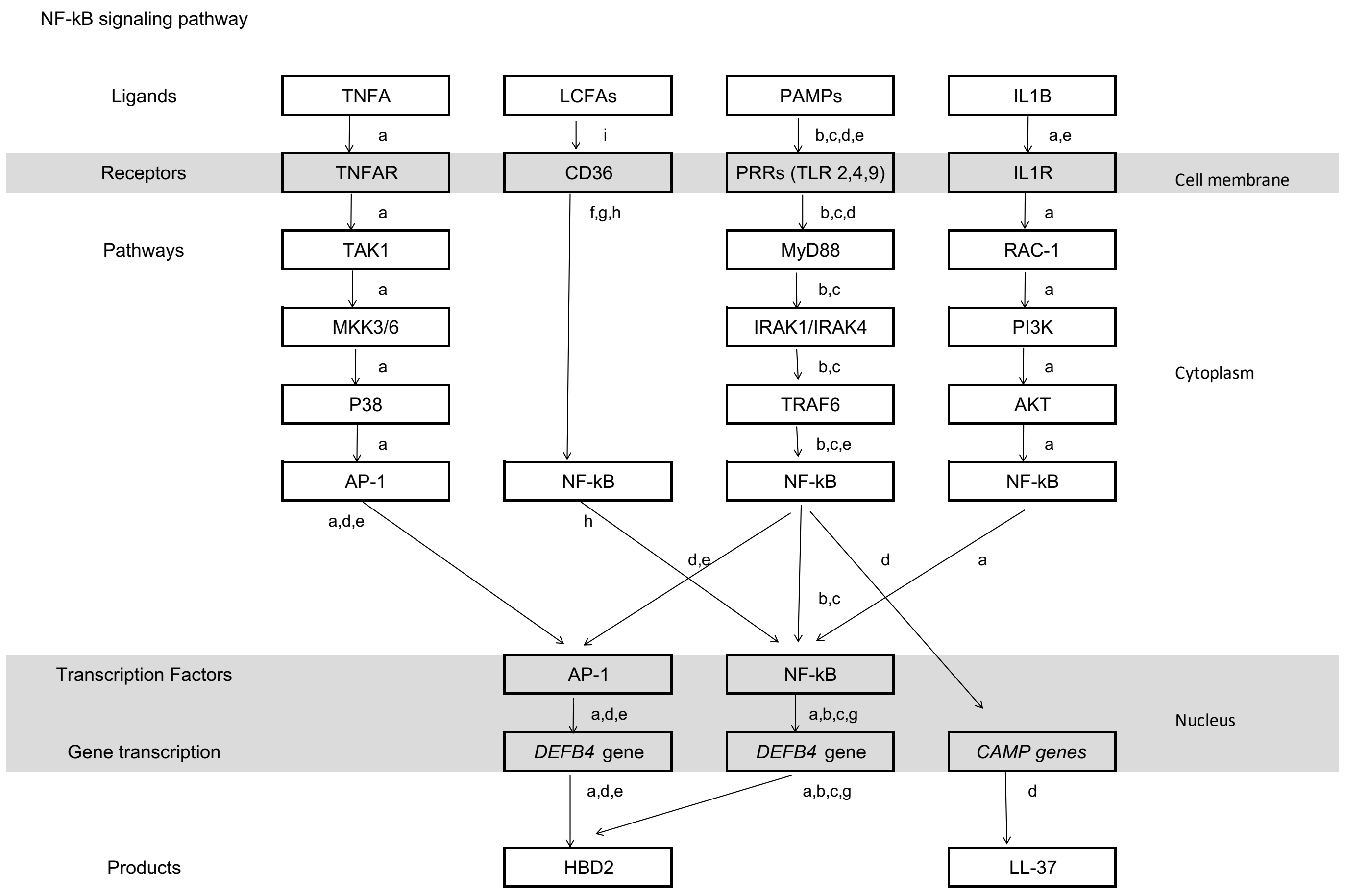

Supplement: Supplementary file 1 [file antibiotics-12-00361-s001.zip › Antibiotics Suppl Fig S3 (02-06-23).pdf]
