# Supplementary material for: Induction of Endogenous Antimicrobial Peptides to Prevent or Treat Oral Infection and Inflammation
Source: Antibiotics (Basel). 2023 Feb 9;12(2):361. doi: 10.3390/antibiotics12020361 (PMC9952314; doi:10.3390/antibiotics12020361)

antibiotics

**Supplemental Fig S4**. Butyrate (BA) and phenylbutyrate (PBA) inhibition of histone deacetylase (HDAC). A scheme showing pathways leading to BA and PBA induced antimicrobial peptide (AMP) transcription and expression by inhibition of HDAC. In this pathway, BA and PBA inhibits HDAC. This activates BDR4, which starts a cascade through LIFR, and JAK and ends by activating transcription factor STAT3. STAT3 activation then likely leads to *DEFB4B* transcription and HBD2 expression. Pathways leading to induction of AMP transcription and HBD2 expression were compiled from results reported in studies by a) Ren et al. 2021 [199], b) Li et al. 2015 [200], and c) Sinha and Yen 2018 [201].

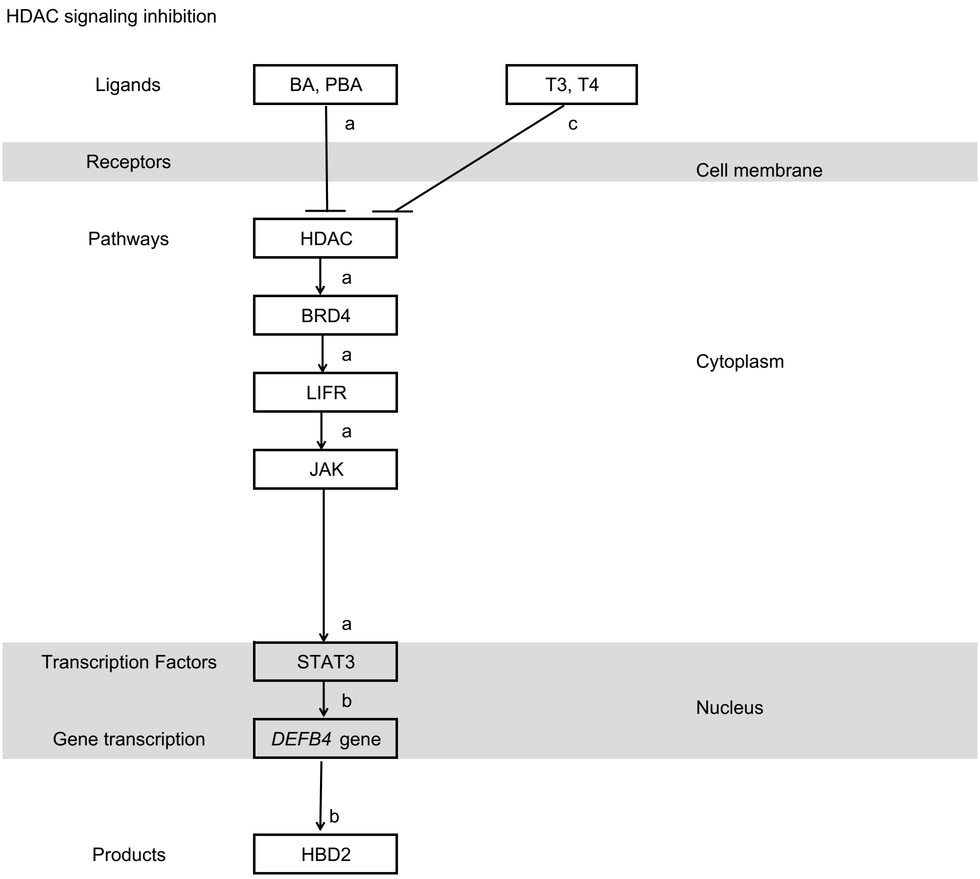

Supplement: Supplementary file 1 [file antibiotics-12-00361-s001.zip › Antibiotics Suppl Fig S4 (02-06-23).pdf]
