# Supplementary material for: Induction of Endogenous Antimicrobial Peptides to Prevent or Treat Oral Infection and Inflammation
Source: Antibiotics (Basel). 2023 Feb 9;12(2):361. doi: 10.3390/antibiotics12020361 (PMC9952314; doi:10.3390/antibiotics12020361)
Supplement: Supplementary file 1 [file antibiotics-12-00361-s001.zip › Antibiotics Suppl Table S1 (02-06-23).pdf]

Supplemental Table S1. Many of the roles antimicrobial peptides (AMPs) play in inflammation; innate and adaptive immunity; angiogenesis, vasculogenesis, and wound healing; and pain antinociception.

AMP	µg/ml	µM	Specific activity (by category)
**Roles of AMPs in inflammation**			
LL-37	0.1	0.02	Binds microbial antigens: a 37-mer of human CAP187 binds to lipopolysaccharide (LPS) [169]
HBD3	1.0	0.19	Binds microbial antigens: HBD3 binds to putative binding sites on *P. gingivalis* HagB [164]
HBD1	1.0	0.25	Binds microbial antigens: HBD1 binds to putative binding sites on *Porphyromonas gingivalis* hemagglutinin B (HagB) [164]
Histatin 5	1.0	0.30	Binds microbial antigens: Histatin 5 binds to putative binding sites on *P. gingivalis* HagB [164]
HBD3	10.0	1.94	Attenuated pathway signaling: HBD3 attenuated phosphorylated ERK1/2 signals to microbial agonist [163]
LL-37	20.0	4.45	Attenuated expression of genes: LL-37 suppressed LPS-induced genes in human monocytes [172]
HBD1	1.0	0.25	Attenuated cytokine response: HBD1 induced lower IL6 and KC responses to rHagB in nasal wash fluid of mice than those given rHagB alone [202]
HBD3	1.0	0.19	Attenuated cytokine response: HBD3 attenuated proinflammatory cytokine responses to co-administered agonists [163]
HBD3	1.0	0.19	Attenuated cytokine response: HBD3 induced lower IL6 and KC responses to rHagB in nasal wash fluid of mice than those given rHagB alone [202]
HBD3	1.0	0.19	Attenuated cytokine response: HBD3 modulated cytokine responses to a microbial antigen [203]
HNP-1	1.0	0.29	Attenuated cytokine response: HNP-1 induced lower KC responses to rHagB in nasal wash fluid of mice than those given rHagB alone [202]
HNP-2	1.0	0.29	Attenuated cytokine response: HNP-2 induced lower IL6 and KC responses to rHagB in nasal wash fluid of mice than those given rHagB alone [202]
LL-37	1.0	0.22	Attenuated cytokine response: 1-15 ug/ml LL-37 attenuated LTA-induced expression of TNF-α in RAW264.7 cells and primary mouse macrophages [204]
LL-37	1.0	0.22	Attenuated cytokine response: LL-37 attenuated LPS-induced expression of TNF-α in THP-1 cells [172]
LL-37	11.3	2.50	Attenuated cytokine response: LL-37 attenuated LPS-induced expression of chemokines and cytokines in gingival fibroblasts [205]
LL-37	5.0	1.11	Activated pathway signaling: LL-37 activated EGFR signaling in HCEC [206]
HNP-1-3	8.0	2.32	Enhanced gene expression: HNP-1-3 cause an increase in NCI-H292 human lung cells mucin gene expression [207]
HBD1	0.1	0.03	Enhanced cytokine response: HBD1 increased GM-CSF in gingival keratinocytes [208]
HBD1	20.0	5.08	Enhanced cytokine response: HBD1 induced the production of CCBMs [155]
HBD2	2.0	0.46	Enhanced cytokine response: 2.0-32.0 ug/ml HBD2 induced production of IL6, IL8, and IL10 from human PBMCs [155]
HBD2	5.0	1.15	Enhanced cytokine response: HBD2 increased IL6 and IL8 in gingival keratinocytes [208]

HBD2	10.0	2.31	Enhanced cytokine response: HBD2, HBD3, HBD4, and LL-37 induced IL18 transcription or IL18 expression from human keratinocytes [209]
HBD2	20.0	4.61	Enhanced cytokine response: HBD2 induced the production of CCBMs [155]
HBD3	5.0	0.97	Enhanced cytokine response: HBD3 increased IL-1β in gingival keratinocytes [208]
HBD3	10.0	1.94	Enhanced cytokine response: HBD2, HBD3, HBD4, and LL-37 Induced IL18 transcription or IL18 expression from human keratinocytes [209]
HBD3	10.1	2.00	Enhanced cytokine response: HBD3 modulated cytokine responses to a microbial antigen [203]
HBD3	20.0	3.87	Enhanced cytokine response: HBD3 induced the production of CCBMs [155]
HNP-1-3	3.0	0.87	Enhanced cytokine response: HNP-1-3-induced IL8 release from A549 airway cells [210]
HNP-1-3	100.0	29.00	Enhanced cytokine response: HNP-1 induced cytokine expression by primary bronchial epithelial cells (PBEC) [211]
HNP-1-3	100.0	29.00	Enhanced cytokine response: HNP-1-3 induced cytokine expression by A549 cells [211]
LL-37	0.5	0.11	Enhanced cytokine response: LL-37 induced heparin-binding EGF-like growth factor release in THCE cells [206]
LL-37	5.0	1.11	Enhanced cytokine response: LL-37 induced heparin-binding EGF-like growth factor release in THCE cells [206]
LL-37	10.0	2.22	Enhanced cytokine response: HBD2, HBD3, HBD4, and LL-37 Induced IL18 transcription or expression from human keratinocytes [209]
LL-37	15	3.34	Enhanced cytokine response: Over 15 ug/ml LL-37 caused cell damage and increased TNF-α expression in RAW264.7 cells and primary mouse macrophages [204]
LL-37	50.0	11.12	Enhanced cytokine response: LL-37-derived mature dendritic cells produce a characteristic Th-1-inducing cytokine profile
**Roles of AMPs in innate immunity**			
HNP-1		0.01	Chemotactic activity: HNP-1 was chemotactic for monocytes [212]
HNP-1-3	100.0	29.00	Chemotactic activity: HNP-1 was chemotactic for A549 cells and primary bronchial epithelial cells (PBEC) [211]
HNP-1-3	100.0	29.00	Chemotactic activity: HNP-1-3 was chemotactic for A549 cells and primary bronchial epithelial cells (PBEC) [211]
HNP-2		0.01	Chemotactic activity: HNP-2 was chemotactic for monocytes [212]
LL-37	1.0	0.22	Chemotactic activity: LL-37 was chemotactic for NCI-HJ292 bronchial epithelial cells [213]
LL-37	5.0	1.11	Chemotactic activity: LL-37 was chemotactic for mast cells [214]
LL-37	5.0	1.11	Chemotactic activity: 5.0-20.0 ug/ml LL-37 was chemotactic for NCI-HJ292 bronchial epithelial cells [213]
LL-37	20.0	4.45	Chemotactic activity: 5.0-20.0 ug/ml LL-37 was chemotactic for NCI-HJ292 bronchial epithelial cells [213]
LL-37	4.5	1.00	Promoted cell migration: LL-37 promoted migration of gingival fibroblasts [205]
LL-37	4.5	1.00	Promoted cell migration: LL-37 promoted proliferation of gingival fibroblasts [205]
HNP-1-3	8.0	2.32	Promoted cell migration: HNP-1-3 increase in NCI-H292 human lung cell migration [207]
LL-37	50.0	11.12	Increased cell markers: LL-37-derived mature dendritic cells had increased expression of CD makers on the surface of immature dendritic cells [179]

HBD1	5.0	1.27	Induced proliferation: HBD1 induced the proliferation of PLE cells [215]
HBD2	5.0	1.15	Induced proliferation: HBD2 induced endothelial cell proliferation [216]
HBD2	5.0	1.15	Induced proliferation: HBD2 induced the proliferation of epithelial cells, KB, SCC-9 and PLE [215]
HBD2	10.0	2.31	Induced proliferation: HBD2 induced endothelial cell proliferation [216]
HBD3	5.0	0.97	Induced proliferation: HBD3 induced epithelial cell and fibroblast proliferation [215]
HBD3	8.0	1.55	Induced proliferation: HBD3 induced fibroblast proliferation [215]
HNP-1	8.0	2.32	Induced proliferation: 5-8 μg/ml HNP-1 induced proliferation of epithelial cells [215]
HNP-1-3	1.0	0.29	Induced proliferation: HNP induced proliferation and T helper cytokine expression profiles of CD4+ T-cells [217]
HNP-1-3	1.0	0.29	Induced proliferation: HNP-1 induced PLE oral epithelial cell proliferation [215]
HNP-1-3	4.0	1.16	Induced proliferation: 4.0-10.0 ug/ml HNPs induced proliferation of A549 human lung cells [218]
HNP-1-3	5.0	1.45	Induced proliferation: HNP induced epithelial cell proliferation [215]
HNP-1-3	5.0	1.45	Induced proliferation: HNP-1 induced primary epithelial cell proliferation [215]
HNP-1-3	8.0	2.32	Induced proliferation: HNP-1 induced KB cell proliferation [215]
HNP-1-3	8.0	2.32	Induced proliferation: HNP-1 induced primary epithelial cell proliferation [215]
HNP-1-3	10.0	2.90	Induced proliferation: 4.0-10.0 ug/ml HNPs induced proliferation of A549 human lung cells [218]
LL-37	1.0	0.22	Induced proliferation: LL-37 induced NCI-H292 bronchial epithelial cell proliferation [213]
LL-37	5.0	1.11	Induced proliferation: LL-37 induced NCI-H292 bronchial epithelial cell proliferation [213]
LL-37	10.0	2.22	Induced proliferation: LL-37 induced NCI-H292 bronchial epithelial cell proliferation [213]
LL-37	20.0	4.45	Induced proliferation: LL-37 induced NCI-H292 bronchial epithelial cell proliferation [213]
LL-37	50.0	11.12	Induced proliferation: LL-37-derived mature dendritic cells induced IFN-γ-secreting T-cell proliferation
LL-37	4.5	1.00	Suppressed apoptosis: LL-37 suppressed apoptosis in keratinocytes [219]
HBD1	10.0	2.54	Decrease in cell number: HBD1 decreased the number of fibroblasts [215]
HBD1	50.0	12.70	Decrease in cell number: HBD1 caused a decrease in KB cell number [215]
HBD2	30.0	6.92	Cell cytotoxicity: HBD2 decreased KB cell number [215]
HNP-1-3	50.0	14.50	Cell cytotoxicity: HNP-1 was cytotoxic for epithelial cells and fibroblasts [215]
HNP-1-3	50.0	14.50	Cell cytotoxicity: HNPs were cytotoxic to A549 human lung cells [218]
LL-37	10.0	2.22	Cell cytotoxicity: LL-37 was cytotoxic for HCECs [206]
LL-37	11.3	2.50	Cell cytotoxicity: 11.3-45.0 ug/ml LL-37 was cytotoxic to gingival fibroblasts [205]
LL-37	20.0	4.45	Cell cytotoxicity: 20, 50 μg/ml LL-37 was cytotoxic for NCI-H292 bronchial epithelial cells [213]
LL-37	45.0	10.01	Cell cytotoxicity: 11.3-45.0 ug/ml LL-37 was cytotoxic to gingival fibroblasts [205]
LL-37	50.0	11.12	Cell cytotoxicity: 20, 50 μg/ml LL-37 was cytotoxic for primary airway epithelial cells [213]
**Roles of AMPs in adaptive immunity**			
LL-37	50.0	11.12	Induced cell differentiation: LL-37 treated dendritic cells were larger with more surface filopodia [179]

LL-37	50.0	11.12	Increased antigen uptake: LL-37 treated dendritic cells bound and took up more FITC-labeled dextran [179]
LL-37	50.0	11.12	Increased co-stimulatory molecule expression: LL-37 increased the expression of CD11b, CD86, and CD83 [179]
LL-37	50.0	11.12	Enhanced Th1 cytokine response: LL-37 treated dendritic cells exposed to LPS produced a T helper type 1 (TH1) cell inducing cytokine profile [179]
HBD1	1.0	0.25	Enhanced antibody response: HBD1 induced higher OVA-specific serum IgG and higher OVA-specific serum IgM in mice [220]
HBD2	1.0	0.23	Enhanced antibody response: HBD2 induced higher OVA-specific serum IgG, lower serum IgM in mice [220]
HBD3	1.0	0.19	Enhanced antibody response: HBD3 induced higher rHagB-specific serum IgG in mice than those immunized with rHagB alone [202]
HNP-1	1.0	0.29	Enhanced antibody response: HNP-1 induced higher OVA-specific serum IgG and lower OVA-specific serum IgM in mice [220]
HNP-1	1.0	0.29	Enhanced antibody response: HNP-1 induced higher rHagB-specific serum IgG in mice than those immunized with rHagB alone [202]
HNP-1-3	1.0	0.29	Enhanced antibody response: HNP enhanced IgG and IgM antibody responses to co-administered antigen [217]
HNP-2	1.0	0.29	Enhanced antibody response: HNP-2 induced low OVA-specific serum IgG and higher OVA-specific serum IgM in mice [220]
HNP-1-3	5.0	1.45	Enhanced antibody response: HNP enhanced IgG and IgM antibody responses to co-administered antigen [217]
HNP-1-3	50.0	14.50	No cell number increase: HNP-1-3 did not cause an increase in NCI-H292 human lung cell mucin gene expression [207]
HBD3	50.0	9.69	No cytotoxicity: HBD3 was not cytotoxic to epithelial cells, KB, SCC-9 and PLE [215]
LL-37	50.0	11.12	No cytotoxicity: LL-37 was not cytotoxic to immature dendritic cells [179]
**Roles of AMPs in angiogenesis, vasculogenesis, and wound healing**			
HBD2	0.5	0.12	Angiogenesis: HBD2 induced the formation of endothelial tubes and capillary-like structures [216]
HBD3	200.0	58.00	Angiogenesis: HBD3 enhanced fibroblast accumulation *in vivo* and migration and proliferation *in vitro* [178]
HBD3	200.0	58.00	Angiogenesis: HBD3 increased transcription of angiogenic growth factors *bfgf*, *pdgf*, and *vegf* [178]
HBD3	200.0	58.00	Angiogenesis: wounds treated with HBD3 increased new vessel formation [178]
HNP-1-3	50.0	14.50	Delayed wound closure: HNP-1-3 delayed wound closure of NCI-H292 human lung cells [207]
HBD2	0.5	0.12	Enhanced wound closure: HBD2 increased the speed of HUVEC wound closure [216]
HBD3	200	58.00	Enhanced wound closure: HBD3 was the most effective concentration for accelerating wound healing in mice [178]
HNP-1-3	4.0	1.16	Enhanced wound closure: 4-10 µg/ml HNP-1-3 enhanced wound closure in NCI-H292 human lung cells [207]

HNP-1-3	10.0	2.90	Enhanced wound closure: 4-10 μg/ml HNP-1-3 enhanced wound closure in NCI-H292 human lung cells [207]
HNP-1-3	8.0	2.32	Enhanced wound closure: 4-10 μg/ml HNPs enhanced repair in mechanically wounded NCI-H292 cell monolayers [207]
LL-37	0.5	0.11	Enhanced wound closure: LL-37 enhanced epithelial wound healing [206]
LL-37	5.0	1.11	Enhanced wound closure: LL-37 enhanced closure in scraped primary airway epithelial cell cultures [213]
LL-37	11.3	2.50	Enhanced wound closure: LL-37 induced antifibrotic activity beneficial for wound healing without fibrosis [205]
HBD2	0.5	0.12	Promoted cell migration: HBD2 induced the migration of HUVEC [216]
HBD2	10	2.31	Promoted cell migration: HBD2 induced the migration of keratinocytes [152]
HBD3	0.5	0.11	Promoted cell migration: HBD3 induced the migration and proliferation of human fibroblasts [178]
HBD3	5	0.97	Promoted cell migration: HBD3 induced the migration of keratinocytes [152]
HBD4	10	2.22	Promoted cell migration: HBD4 induced the migration of keratinocytes [152]
HBD3	200	58.00	Enhanced wound healing: HBD3 was the most effective concentration for accelerating wound healing in mice [178]
**Roles of AMPs in pain nociception**			
PCD-1	20.0	3.69	Pain antinociception: 20 ug PCD-1 was injected (intrathecal injection) into rats and significantly inhibited chronic constriction injury-induced thermal hyperalgesia, mechanical allodynia, cold allodynia, and weight-bearing deficits [189]
Ueq 12-1	0.2 mg/kg	Not applicable	Pain antinociception: 0.2 mg/kg Ueq 12-1 was injected (i.v.) into mice and reduced allyl isothiocyanate -induced paw licking, guarding, and paw edema and reduced Freund's complete Adjuvant-induced thermal hyperalgesia and paw edema [188]
Alloferon	0.1265	0.10	Pain antinociception: 100 nM Alloferon 1 was injected (i.c.v.) through implanted cannulas in the right lateral brain ventricle of rats and induced significant antinociceptive effects in the tail immersion and the hot plate tests of pain [186]
CgA	0.5 mg/kg	Not applicable	Pain antinociception: 0.5 mg/kg CgA 47–66 was injected (i.p.) into rats and induced maximal antinociceptive effects in their responses in the acetic acid-induced writhes test of pain [192]
AMV	6 mg/kg	Not applicable	Pain antinociception: 50 or 100 pg *Apis mellifera* venom (AMV) in 20 μl was injected into foot pads of mice and induced antinociceptive effects in their responses in the hot-plate pain tests of pain [187]
