# Supplementary material for: Induction of Endogenous Antimicrobial Peptides to Prevent or Treat Oral Infection and Inflammation
Source: Antibiotics (Basel). 2023 Feb 9;12(2):361. doi: 10.3390/antibiotics12020361 (PMC9952314; doi:10.3390/antibiotics12020361)
Supplement: Supplementary file 1 [file antibiotics-12-00361-s001.zip › Antibiotics Suppl Table S2 (02-06-23).pdf]

antibiotics

MDPI

Supplemental Table S2. Irradiation induction of antimicrobial peptides (AMPs). Examples of irradiation regulated AMP transcription and expression at different wavelengths, doses, and minimal erythema doses (MED) in keratinocytes, skin explants, and human skin. The outcomes are often dependent upon the cell type, the wavelength used, the dose used, and the time of treatment.

**Wavelength dose, or MED**	**Cells/tissues**	**AMP transcription and expression [reference]**
**UVC (280-100 nm)**		
254 nm; 6.38 J/m²	Keratinocytes	*CAMP* and *DEFB1* expression were increased after exposure [56]
**UVB (315-280 nm)**		
290-320 nm; emission peak 313 nm; 350 J/m²	Keratinocytes	*DEFB4B*, *DEFB103A*, *RNASE7*, and *S100A7* transcription and HBD2, HBD3, RNase7, and S100A7 expression were increased [100]
280-320 nm; emission peak 313 nm; 30, 100 mJ/cm²	Keratinocytes	HBD1 and HBD2 expression were increased in HaCaT cells [96]
290-320 nm; emission peak 313 nm; 0, 150, 350 J/m²	Keratinocytes	*DEFB4B*, *DEFB103A*, *RNASE7*, and *S100A7* transcription and HBD2, HBD3, RNase7, and S100A7 expression were increased [100]
365 nm; 6.38 J/M²	Keratinocytes	*DEFB1* expression was increased 5 minutes after exposure, but not 10 and 20 minutes after exposure [56]
290-320 nm; emission peak 313 nm 3 X 1 MED	Keratinocytes	*DEFB4B*, *DEFB103A*, *RNASE7*, and *S100A7* transcription and HBD2, HBD3, RNase7, and S100A7 expression were increased [100]
280-320 nm; emission peak 313 nm; 20 mJ/cm²	Keratinocytes	*DEFB4B* and *CAMP* transcription and HBD2 and LL-37 expression were increased [95]
280-320 nm; emission peak 313 nm; 20 mJ/cm²	Keratinocytes	*CAMP* transcription and LL-37 expression was increased [95]
290-320 nm; emission peak 313 nm; 250 J/m²	Skin explants	*DEFB103A*, *RNASE7*, and *S100A7* transcription and HBD3, RNAse7, and S100A7 expression were increased [100]
280-315 nm; 1 MED	Healthy skin	hCAP18 and vitamin D receptor expression were increased [102]
310-315 nm; emission peak 311 nm; 1.6 J/cm² total	Atopic eczema	HBD1 expression was increased and HBD2 expression was decreased compared to healthy controls [146]
312 nm; 2 MED	Skin biopsies	*DEFB4B*, *PI3*, *S100A7*, *S100A8/9*, and *S100A12* transcription and HBD2, PI3, S100A7, S100A8/9, and S100A12 expression were increased [139]
**UVA (340-400 nm)**		
365 nm; 6.38 J/m²	Keratinocytes	*DEFB1* expression was increased 5 minutes after exposure [56]
340-400 nm; 20 J/cm²; cumulative dose 800 J/cm²	Localized scleroderma	HBD1 and HBVD3 expression were decreased compared to normal skin and HBD3 expression was increased in normal skin [147]
**Green light (520–530 nm)**		
525 nm; 15 mW/cm²	Fibroblasts	LL-37 and HBD1 expression were increased in infected oral fibroblasts [103]
**Red light (625-740 nm)**		
625 nm; 15 mW/cm²	Fibroblasts	LL-37 and HBD1 expression were increased in infected oral fibroblasts [103]
**NIR (near infrared irradiation, 800-2,500 nm)**		
810 nm; 4 J/cm²	Fibroblasts	HBD2 expression was increased in oral fibroblasts [104]
