# Supplementary material for: Induction of Endogenous Antimicrobial Peptides to Prevent or Treat Oral Infection and Inflammation
Source: Antibiotics (Basel). 2023 Feb 9;12(2):361. doi: 10.3390/antibiotics12020361 (PMC9952314; doi:10.3390/antibiotics12020361)
Supplement: Supplementary file 1 [file antibiotics-12-00361-s001.zip › Antibiotics Suppl Table S3 (02-06-23).pdf]

**Supplemental Table S3.** Synergistic activity of molecules on induction of antimicrobial peptides (AMPs). Examples of synergistic activity of micronutrients (vitamin D), nutrients and macronutrients (isoleucine, lactose, grains, butyrate (BA), and phenylbutyrate (PBA)), and proinflammatory cytokines (IL-1β, TNF-α, and IFN-γ) on AMP transcription and expression.

| Synergistic activity | AMP transcription and expression | Reference |
|---|---|---|
| Lactose, BA | Increased *CAMP* transcription | [72] |
| Lactose, PBA | Increased *CAMP* transcription | [72] |
| Lactose, PBA | Increased LL-37 expression in HT-29 cells | [86] |
| PBA, vitamin D3[a] | Increased *CAMP* transcription and LL-37 expression | [87] |
| Cyclic AMP, BA | Increased avian beta-defensin (AvBD) 9 expression | [82] |
| Vitamin D3[a], IL13 | IL13 induced vitamin D-dependent hCAP18/LL-37 expression | [221] |
| IL-1β, TNF-α | Increased *DEFB4B* transcription | [98] |
| IL-1β, IFN-γ | Increased DEFB4B and *DEFB103A* transcription | [98] |
| IFN-γ, TNF-α | Increased *DEFB103A* transcription | [98] |
| IL-1α and Isoleucine | IL-1α and isoleucine increased *DEFB4B* transcription in Caco2 cells | [77] |
| Grains, *Escherichia coli* | Amaranth, millet, soybean, and sesame flours boosted *DEFB4B* transcription with *E. coli* | [12] |

[a] 1,25-Dihydroxvitamin D3; 1,25$(OH)_2$D3
